# Broad-spectrum antimicrobial activity of wetland-derived *Streptomyces* sp. ActiF450

**DOI:** 10.17179/excli2020-1124

**Published:** 2020-03-12

**Authors:** Mabrouka Benhadj, Roumaisa Metrouh, Taha Menasria, Djamila Gacemi-Kirane, Fatma Zohra Slim, Stephane Ranque

**Affiliations:** 1 Department of Applied Biology, Faculty of Exact Sciences and Natural and Life Sciences, Larbi Tebessi University, 12002 Tebessa, Algeria; 2 Biomolecules and Application Laboratory, Faculty of Exact Sciences and Natural and Life Sciences, Larbi Tebessi University, 12002 Tebessa, Algeria; 3 Department of Biochemistry, Faculty of Science, University Badji Mokhtar Annaba, Annaba, 23000, Algeria; 4 Aix Marseille University, IRD, APHM, SSA, VITROME, IHU-Méditerranée Infection, 19-21 Boulevard Jean Moulin, 13005 Marseille, France

**Keywords:** coastal wetland, Streptomyces, antifungal activity, Candida spp.

## Abstract

The increased incidence of invasive infections and the emerging problem of drug resistance particularly for commonly used molecules have prompted investigations for new, safe and more effective microbial agents. Actinomycetes from unexplored habitats appear as a promising source for novel bioactive compounds with a broad range of biological activities. Thus, the present study aimed to isolate effective wetland-derived actinomycetes against major pathogenic fungi and bacteria. Water samples were collected from various locations of Fetzara Lake, Algeria. Thereafter, an actinomycete designated ActiF450 was isolated using starch-casein-agar medium. The antimicrobial potential of the newly isolated actinomycete was screened using the conventional agar cylinders method on Potato Dextrose Agar (PDA) against various fungal and bacterial pathogens. A wetland-derived *Streptomyces* sp. Actif450 was identified as *Streptomyces* *malaysiensis* based on its physiological properties, morphological characteristics, and 16S rDNA gene sequence analysis. The antimicrobial activity of *Streptomyces* sp. ActiF450 showed a potent and broad spectrum activity against a range of human fungal pathogens including moulds and yeasts, such as *Arthroderma vanbreuseghemii, Aspergillus fumigatus, A. niger, Candida albicans, C. glabarta, C. krusei, C. parapsilosis, Fusarium oxysporum, F. solani, Microsporum canis, Rhodotorula mucilaginous *and* Scodapulariopsis candida*. In addition, high antibacterial activity was recorded against pathogenic staphylococci. The novel *Streptomyces* sp. ActiF450 may present a promising candidate for the production of new bioactive compounds with broad-spectrum antimicrobial activity.

## Introduction

The last two decades have seen unprecedented changes in the pattern of fungal diseases (FDs) in humans. The rising prevalence of FDs, such as candidiasis, aspergillosis, pneumocytosis and cryptococcosis, have become a major health problem worldwide (Casadevall, 2018[13]; Richardson and Warnock, 2012[33]). 

Fungal diseases are difficult to manage because they tend to be chronic, hard to diagnose, and more recalcitrant to therapy such that most mycoses require treatment courses lasting months or longer (Liu et al., 2018[24]). These diseases have gained a much greater importance, largely because of their increasing incidence among transplant recipients and immune compromised patients, including those with acquired immunodeficiency syndrome (AIDS) (Richardson and Warnock 2012[33]). In fact, the more widespread use of immunosuppressive therapies and increased movements of patients at risk are among the main acquired risk factors contributing to FDs (Liu et al., 2018[24]).

The world is facing an ever-increasing problem of antimicrobial resistance (Menasria et al., 2015[29]). In fact, new resistance mechanisms emerge and spread globally threatening our ability to treat common infectious diseases, resulting in death and disability of individuals (Boukoucha et al., 2018[12]). Different saprophyte and commensal fungi from both yeast and mold forms are recognized among agents that cause human mycosis (Benammar et al., 2017[7]), including species of *Candida *spp., *Aspergillus *spp., *Pneumocysti *spp., dimorphic (*Coccidioides* and *Paracoccidioides*), dermatophytes (*Trichophyton *spp.), and encapsulated yeast *Cryptococcus *spp., which are present in the localized and disseminated forms of the disease (Liu et al., 2018[24]; Richardson and Warnock, 2012[33]). Fungal infections including superficial and invasive fungal infections 'IFIs' have increased significantly during the past last decades, which can be an economical burden and a substantial medical concern, particularly in immunocompromised population (Antinori et al., 2018[4]). Also, the effectiveness of current antifungal therapies in the management of these infections is under discussion, due to several limitations, such as off-target toxicity and drug-resistant emergence (Liu et al., 2018[24]).

The increased incidence of invasive mycoses and the emerging problem of drug resistance (Casadevall, 2018[13]), particularly for the azole and polyene compounds or commonly used molecules, have prompted investigations for new, safe and more effective antifungal agents (Liu et al,. 2018[24]). In addition, the incidence of *S. aureus*-linked infections has increased, with highly virulent strains being encountered (Menasria et al., 2015[29]). The aforementioned pathogens can be acquired from hospitals and increasingly from non-clinical environments (i.e., community-associated infections) (Antinori et al., 2018[4]; Merradi et al., 2019[30]).

Microorganisms are known to produce various bioactive compounds with great potential to be developed as therapeutic drugs for humans and animal uses (Chaudhary et al., 2013[14]). In fact, many of these compounds were derived from the genus *Streptomyces *(Benhadj and Gacemi-Kirane, 2016[8])*. *So far, over 850 species of *Streptomyces* have been isolated and validly published (LPSN, 2019[25]), and more than 600 species have been recorded to be excellent sources of bioactive molecules (Supong et al., 2017[37]).

*Streptomyces* are generally prevalent in soils and diverse natural habitats (Seipke et al., 2012[36]). It is known to be the largest antibiotic-producing genus in the microbial world discovered so far (Benhadj et al., 2019[10]), also having the ability to produce other important bioactive secondary metabolites, such as antifungals, antivirals, antitumorals, anti-hypertensives, and especially immuno-suppressants (de Lima Procópio et al., 2012[17]). Accumulated evidence indicates that the production of bioactive compounds from actinomycetes is associated with nonribosomal peptide synthetase (NRPS) and polyketide synthase (PKS) pathways (Albright et al., 2014[2]), suggesting that nonribosomal peptides, polyketides and their hybrid compounds are the major secondary metabolites of actinomycetes (Komaki et al., 2018[21]).

Sequencing of *Streptomyces *genomes has shown treasure troves of unsuspected and uncharacterized biosynthetic gene clusters, referred as silent or cryptic pathways, for secondary metabolites and antibiotic-like substances than originally anticipated (Niu et al., 2016[31]). These cryptic gene clusters are substantially tied to the environmental conditions in which secondary metabolites production may evolve (Onaka, 2017[32]). Generally, studies on actinobacteria are confined to the terrestrial ecosystems and less significance has given to marine or fresh water systems. It has been shown that marine actinomycetes and strains from unexplored habitats were found to represent a rich source for diverse bioactive metabolites with potential applications (Benhadj et al., 2018[10]).

Algeria harbors several wetlands of which fifty are classified as Ramsar sites of international importance in terms of biodiversity and functional role (Menasria et al., 2019[28]). However, aspects related to microbiota remain little known. It is suggested that the changes in salinity, light, temperature, nutrient availability and other physicochemical and microbiological processes in such ecosystem turned out to be the driving forces for metabolic pathway adaptations that could result in the production of valuable metabolites (Menasria et al., 2018[27]). In this study, a potent antimicrobial actinobacteria was isolated from a coastal wetland (Ramsar wetland) within the West Mediterranean Basin (Fetzara Lake, northeastern Algeria) (www.ramsar.org). This strain was identified as *Streptomyces* sp. ActiF450 and exhibits broad antifungal spectrum activities against different medically important bacteria (*Satphylococcus aureus*), yeast and moulds species like *Candida *spp*., Kluveromyces* spp., *Rhodotorula *spp.,* Aspergillus *spp.,* Fusarium *spp., *Microsporum *spp., and others. In addition, to evaluate the antimicrobial potency, various extractions of solid cultures using different organic solvents were performed.

## Material and Methods

### Sample collection and isolation 

Water samples from Fetzara Lake (36°43' and 36°50'N, 7°24' and 7°39'E) were collected during winter (January 2017). The samples were heat treated at 50 °C for 30 min and 10-fold serial dilutions were prepared. Aliquots of 0.1 ml were spread plated onto Casein Starch agar (CSA) (g/l) (soluble starch, 10; casein, 0.3; KNO_3_, 2.0; MgSO_4_.7H_2_O, 0.05; K_2_HPO_4_, 2.0; NaCl, 2; CaCO_3_, 0.02; FeSO_4_.7H_2_O, 0.01 agar 20) supplemented with (2.5 μg/ml of rifampicin, 10 μg/ml of amphotericin B and 75 μg/ml of fuconazol). Plates were incubated at 30 °C for 7 days up to 4 weeks, and actinobacteria-like strain designated ActiF450 was isolated, subcultured and maintained on CSA slants at 4 °C. Spore suspensions were prepared in glycerol 20 % and stored at -80 °C for further use.

### Morphological characteristics of the isolate

Morphological characteristics such as aerial mass color and substrate mycelium were observed on soya flour mannitol (SFM) plates. The aerial mass and color of the substrate mycelium were recorded and classified according to Bergey's Manual of Systematic Bacteriology and ISCC-NBS Color Charts standard (Kelly and Judd, 1964[19]; Vos et al. 2009[40]). The tolerance of NaCl, pH, and the effect of temperature was determined by cultivating on ISP2 medium. Nitrate reduction, hemolytic activity and production of amylase, gelatinase, protease and lipase were determined by cultivation on various media as described by Benhadj et al. (2019[10]) using starch, gelatin, casein and Tween 80 as substrates, respectively.

### DNA extraction, molecular identification and phylogenetic analyses

The genomic DNA of the strain ActiF450 was extracted as described by Kieser et al. (2000[20]). A loopful of mycelium was scraped from colonies grown on CSA and suspended in 5 ml of saline-EDTA (5M NaCl, 0.5 M EDTA [pH 8.0], Tris-HCL [pH 7.5]) by vortexing. Lysozyme was added to a final concentration of 1 mg/ml, followed by incubation at 37 °C for 60 min. Later, 10 μl of 1 % (wt/vol) proteinase K and 200 μl of 10 % sodium dodecyl sulfate were added, and the mixed solution was incubated at 50 °C for 2 hours. Subsequently, 500 μl of 5M NaCL was added and the lysate was centrifuged (15,000 g, 5 min) before sequential extractions with phenol, followed by chloroform. The aqueous phase was precipitated using 0.6 ml isopropanol and two volumes of 70 % ethanol. The DNA was suspended in 100 μl of TE 1X (10 mM Tris-HCl at pH 8, 1 mM EDTA) and stored at -20 °C for further use.

The 16S rRNA gene was amplified by PCR using the universal primers Fd1 (5' AGAGTTTGATCCTGGCTCAG) and rP2 (5'-AAGGAGGTGATCCAGCC) (Weisburg et al., 1991[41]). Sequence similarity calculations were carried out using an alignment search program with the EzTaxon-server (Chun et al., 2007[16]). Evolutionary trees based on the aligned sequences were inferred using the neighbor-joining method (Saitou and Nei, 1987[34]) and topologies were evaluated by bootstrap sampling expressed as percentage of 1000 replicates. Phylogenetic analyses were conducted using MEGA software version 6 (Tamura et al., 2013[38]).

### Test microorganisms

For antifungal activity investigation, the following pathogenic yeasts and filamentous fungi were used: *Candida krusei* ATCC6258*, Candida parapsilosis *ATCC22019, clinical *Candida *isolates (ICF18, ICF19, ICF20, ICF21, ICF22, ICF23, ICF24, ICF35, ICF37 and ICF38),* Saccharomyces* sp. ICF43,* Kluveromyces* sp. ICF44, *Rhodotorula mucilaginosa *YA1, *Aspergillus fumigatus *MA1*, A. calidoustus *A5*, A. niger (*IAF27 and MA2), *Aspergillus* sp. ICF58V, *Arthroderma vanbreuseghemii* ICF62B, *Scopulariopsis brevicaulis* ICF57*, Fusarium oxysporum, F. solani, Scedosporium apiospermum, Lichtheimia corymbifera *ST87*, Lomentospora prolificans *ST67*, Microsporum canis* ICF58B*, Pecilomyces variotii, Penicillium chrysogenum *ICF 59, *Rhizopus oryzae *and *Scodapulariopsis candida *ICF53. For antibacterial activity, seven clinical *Staphylococcus aureus* isolates were used including two references strains *Staphylococcus aureus *ATCC25293 and *Staphylococcus aureus* ATCC43300. All fungal and bacterial cultures were maintained on Potato Dextrose Agar (PDA) and Luria Bertani slants respectively at 4 °C. 

### Antimicrobial activity screening

Antimicrobial activity was first screened using the conventional agar cylinders method on Potato Dextrose Agar (PDA) and Luria Bertani (LB) plates for fungi and bacteria respectively (Benhadj et al., 2019[10]). Mycelium plugs (7 mm diameter) of ActiF450 incubated at different time (3, 7, 10 and 14 days) were inoculated onto PDA and LB plates previously inoculated with target pathogens. Secondly, a double layer method was used for confirmation. The active strain ActiF450 was inoculated as a spot in the center of ISP2 plates at 30 °C for 7 days. After incubation, the plates were then covered by 10 ml of PDA and LB previously inoculated with target fungi and bacteria respectively (Badji et al., 2005[6]). The inhibition zones around each spot were measured (mm) after 24 h at 37 °C for bacteria, 48 h and 7 days at 30 °C for yeast and moulds respectively. Two replicates were prepared for each test and plates with indicator strain were used as control.

### Extraction of bioactive molecules

To extract antimicrobial compounds, strain ActiF450 was inoculated onto ISP2 plates at 30 °C for 10 days. After growth, the cultures were fragmented and extracted with an equal volume of different solvents (n-butanol, dichloromethane, ethanol, ethyl acetate, methanol and hexane). The mixtures were filtered through a Whatman No. 1 filter after a vigorous agitation for one hour, and the collected organic extracts were concentrated using a rotary vacuum evaporator. The crude extracts were separately dissolved in 10 % dimethyl sulfoxide (DMSO) and absolute methanol with a final concentration of 20.0 mg/ml. After filtering through a 0.22 μm Millipore filter, different crude extract solutions were used to analyze the inhibition ability using the disc diffusion method (Mehalaine et al., 2017[26]). 

## Results and Discussion

### Phenotypic and molecular characterization of ActiF450

**Figure 1:**
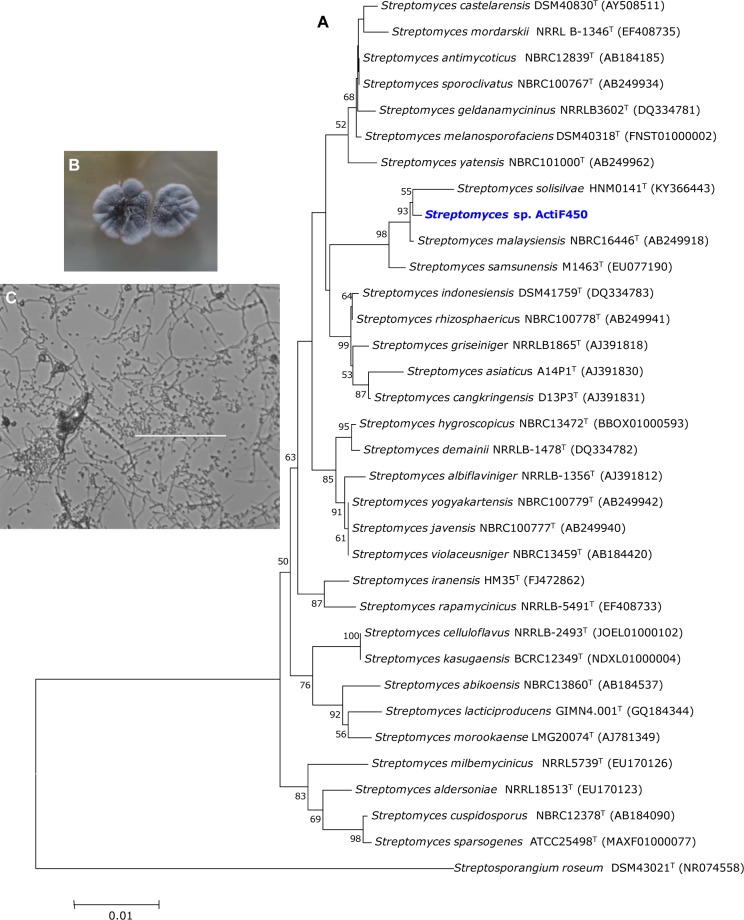
Neighbor-joining tree based on 16S RNA gene sequences of *Streptomyces* sp. ActiF450 and closely related species. Numbers at the nodes indicate levels of bootstrap support based on an analysis of 1000 re-sampled datasets. The scale bar corresponds to 0.01 substitutions per nucleotide position (A). Morphological characters of colony (B), and cell morphology (C) of the strain ActiF450. The strain ActiF450 was grown on SFM medium and the aerial mycelia morphology was viewed using optical microscopy (x100).

In the present study, an active actinobacterium ActiF450 strain was isolated from a natural and unexplored wetland ecosystem (Fetzara Lake) located in Northeastern Algeria. The vegetative and aerial mycelia as well as soluble pigments of the strain ActiF450 were evaluated after cultures on different media for 10 days at 30 °C. Colors of mycelia were determined using the ISCC-NBS centroid color chart (Kelly and Judd, 1964). ActiF450 can grow well on ISP1, ISP2, Bennett and Glucose Yeast Extract Agar (GYEA). The strain produced white grayish aerial and substrate mycelia on ISP2 agar. However, no diffusible pigments were produced, after 10 days of incubation. The microscopic analysis revealed that the strain ActiF450 produced well developed and branched aerial mycelium with spore chains in the top ends (Figure 1(Fig. 1)). The strain was moderately halo tolerant up to 5 % of NaCl concentration. Growth occurs at 25 to 40  °C (optimum, 37  °C) and pH 6.0-9.0 (optimum, pH 7.0). ActiF450 could degrade casein, starch, Tween 80 and liquefy gelatin indicating the variety for their complex metabolites and genomic organization (Bentley et al., 2002[11]). However, the strain was unable to produce urease and H_2_S.

The 16S rRNA partial sequence (1,417 bp) of the strain ActiF450 was amplified and sequenced. The data indicated that strain ActiF450 belongs to the genus *Streptomyces* and was referred to as *Streptomyces* sp. ActiF450. The sequence was subjected to alignment and the BLAST search showed high level of similarity values (99.65 %, 99.22 % and 99.15 %) with *Streptomyces malaysiensis* NBRC16446 ^T ^(Al-Tai et al., 1999[3]), *S. samsunensis *M1463^T^ (Sazak et al., 2011[35]) and *S. solisilvae *HNM0141^T^ (Zhou et al., 2017[43]), respectively. The result was supported by the phylogenetic tree constructed using the neighbor-joining method, where *Streptomyces* sp. ActiF450 formed a subclade and clustered with both* S. malaysiensis *and *S. samsunensis *(Figure 1(Fig. 1)). 

### Antimicrobial activity of ActiF450

The results presented in Table 1(Tab. 1) showed that the strain *Streptomyces* sp. ActiF450 exhibited a broad-spectrum antimicrobial activity against both indicator organisms (bacteria and fungi), from which highly activity were recorded. High inhibitory activities were found against the yeast *Rhodotorula mucilaginosa *YA1 (inhibition zone diameter of 67.6±2.7 mm), and filamentous fungi *Aspergillus niger *MA2 (54.0 ± 6.0 mm), followed by *Microsporum canis* ICF58B (55.0 mm), *Arthroderma vanbreuseghemii* ICF62B (49.2±2.8 mm), *Fusarium oxysporum *F15 (41.2±7.4 mm), *Penicillium chrysogenum *ICF59 (41.1±5.9 mm) and *Scodapulariopsis candida *ICF53 (35.0±2.5 mm). Data clearly indicate that strain ActiF450 exhibited a significant antimicrobial activity against *Candida* spp. (15 to 27 mm), *Candida*-like species *Kluveromyces* sp. ICF44 (25.6±6.2 mm), *Saccharomyces* sp. ICF43 (17.0±0.0 mm)as well as Gram-positive bacteria (*Staphylococcus aureus* clinical isolates) (26.5-35.5 mm). 

**Figure 2:**
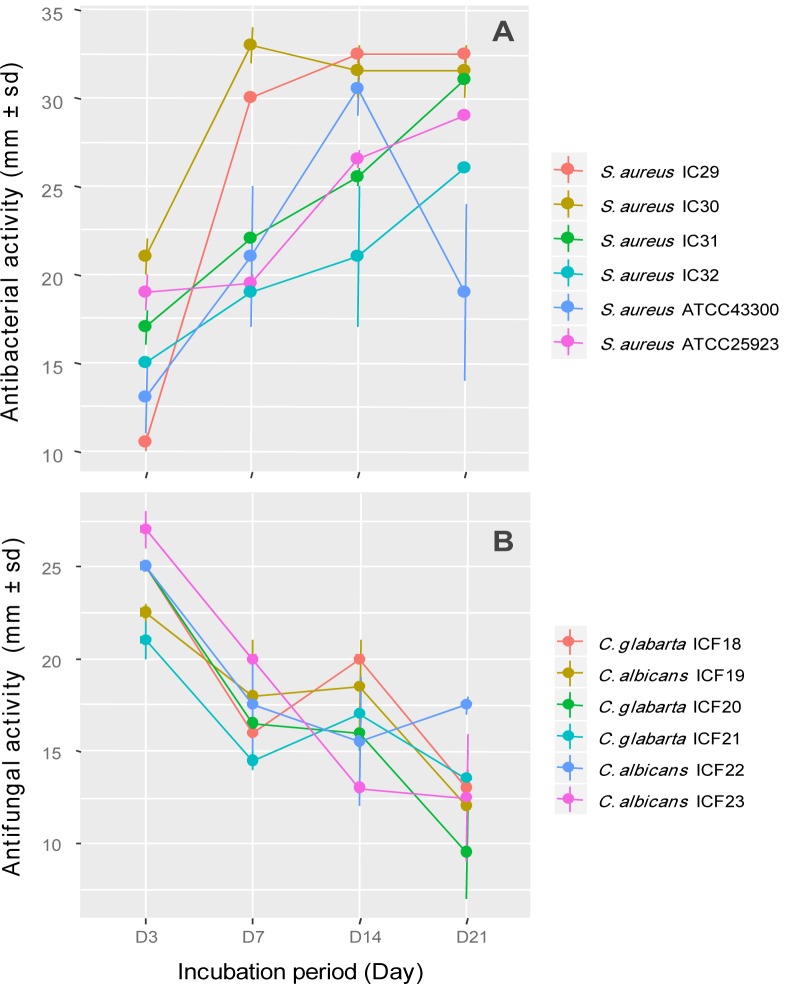
Effect of incubation period on antimicrobial activity of *Streptomyces* sp. ActiF450 against *Staphylococcus* spp. (A) and *Candida* spp. strains (B).

As shown in Figure 2a(Fig. 2), the antimicrobial activity of the strain ActiF450 against selected *S. aureus* isolates started after two days of incubation and reached a maximum after seven days of culture in ISP2. These activities were persistent until the end of the incubation. Furthermore, the anticandidal activity was observed at the first four days of incubation. However, the highest activity was recorded at the third day of incubation period. Thereafter, the activity dramatically declined after one week (Figure 2b(Fig. 2)).

Several reports highlighted the production of various antimicrobial compounds by *Streptomyces *strains with different and variable activities depending on culture conditions including; media nature and/or composition (nitrogen and carbon sources), time of incubation even the indicator organisms used in the confrontation test (Vijayakumar et al., 2012[39]). 

The differences in susceptibility among tested organisms are in concordance with previous studies by the fact that fungal organisms are eukaryotic, and their structures are different from bacteria (Benhadj et al., 2019[10]). The absence of acidic phospholipids and presence of sterols may reduce susceptibility of eukaryotic cells to lytic molecules (Alan and Earle, 2002[1]). The maximum antibacterial activity after seven to ten days of incubation may be attributed to the fact that ActiF450 reached the stationary phase of growth. It has been reported that the bioactive metabolites production by *Streptomyces* takes place in the stationary phase of the growth (Augustine et al., 2004[5]) and the decrease in the anticandidal activity after three days can be attributed to the decrease in the supply of nutrients or the accumulation of toxic substances.

**Figure 3:**
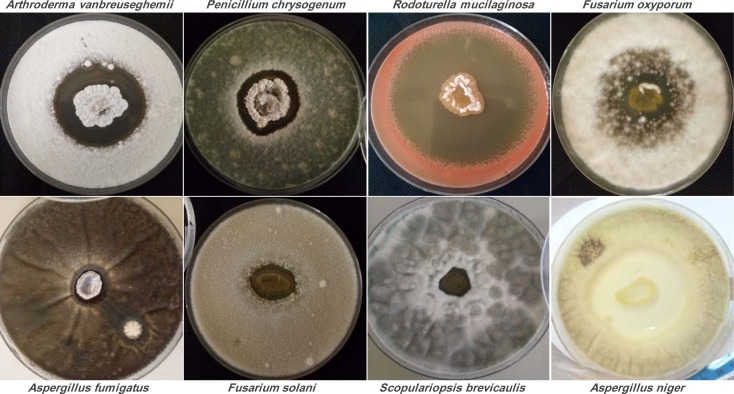
Antifungal activity of *Streptomyces* sp. ActiF450 on the growth of different test organisms. The active strain ActiF450 was inoculated as a spot in the center of ISP2 plates at 30 °C for 7 days. After, the plates were then covered by 10 ml of PDA previously inoculated with target fungi.

Members of the genus *Streptomyces* have attracted extensive attention due to the tremendous success of their natural products in practical application. With their complex life cycle, *Streptomyces *are renowned for the production of an outstandingly large number of bioactive metabolites and accounts for 80 % of the currently available antibiotic-like substances including antifungals and antibacterials (Niu et al., 2016[31]). However, few studies related to the antimicrobial activities of *Streptomyces malaysiensis*, or the closest strains (*S. samsunensis* and *S. solisilvae*) were attempted so far*. *Our results indicate that ActiF450 could be a potential strain for the development of antimicrobial drugs against a wide range of human pathogenic bacteria and fungi including *Candida*-like species and dermatophytes (Figure 3(Fig. 3)).

### Extraction of bioactive molecules

As an effort to study the antimicrobial potential of the strain, assays were conducted on the different extracts of ActiF450 against *Candida albicans* sp. ICF23 and* S. aureus* IC30 (Table 2(Tab. 2)). Traditionally, the discovery of bioactive molecules from microbial sources such as actinomycetes has generally involved, cultivation under different growth conditions, screening of biological activities, extraction of the metabolites, and analysis of the extract for bioactivity. The results reflected that the ethyl acetate, hexane and methanol extracts dissolved in DMSO at 10 % exhibited potent activity against both *C. albicans* ICF23 and *S. aureus *IC30. Other extracts showed moderate activity. In addition, significant differences in susceptibility were noted; where *S. aureus *was high susceptible to ActiF450 extracts compared to *C. albicans*. 

This fact might indicate the existence of a synergistic effect among different metabolites produced by ActiF450 which is lost when they are separated during the extraction (Genilloud, 2017[18]). Otherwise, the antifungal activity might be related to the presence of proteins or enzymes labile to hydrophilic solvents such as chitinases and glucanases capable of degrading the cell wall of the fungi (Benhadj et al., 2019[10]). Studies reviewed by Genilloud (2017[18]) show that actinomycetes continue to be a valuable source of antibiotic activity as they produce a broad spectrum of different antifungal and antibacterial compounds. Moreover, study results indicate a marked effect of the extraction solvents on the isolation of bioactive compounds. It has also been reported that organic solvents provides a higher efficiency in extracting compounds for antimicrobial activities (Lima-Filho et al., 2002[23]).

Previous study on *Streptomyces malaysiensis *MJM1968 reported a strong inhibitory activity *in vitro* by dual-culture *in vitro* assay on several phytopathogenic fungi such as *Alternaria mali, Cladosporium cladosporioides, Colletotrichum gloeosporioides, Fusarium chlamydosporum, Fusarium oxysporum, Rhizoctonia solani* (Cheng et al., 2010[15]). In addition, the compound, which exhibited antifungal activity, was identified as Azalomycin F complex. 

Recently, Zhang et al. (2019[42]) have reported the effect of different fermentation factors for Azalomycin F production from *S. malaysiensis* strain ECO00002. Similarly, Li et al. (2008[22]) have also reported another promising antifungal activity of perhydrofuropyran C-nucleoside named Malayamycin isolated from *Streptomyces malaysiensi*s ATB-11 (Al-Tai et al., 1999[3]). 

## Conclusion

The present study provides evidence that Algerian wetlands are a rich and valuable resource of potentially active actinomycetes. Concisely, this study highlighted for the first time the antimicrobial potentials of wetland-associated actinobacteria with the ability to suppress major human fungal and bacterial pathogens *in vitro*. Further studies are needed to enhance isolation and selection of actinomycetes from unexplored ecosystems for antibiotic discovery. Hopefully, these new agents will meet the challenges as we attempt to manage serious underlying infection diseases. In addition, knowledge of the actinobacteria gene clusters may provide important answers toward understanding the metabolites biosynthetic pathway. 

## Disclosure of interest

The authors declare no conflicts of interest regarding the publication of this study.

## Funding sources

This research did not receive any specific grant from funding agencies in the public, commercial, or not-for-profit sectors.

## Figures and Tables

**Table 1:**
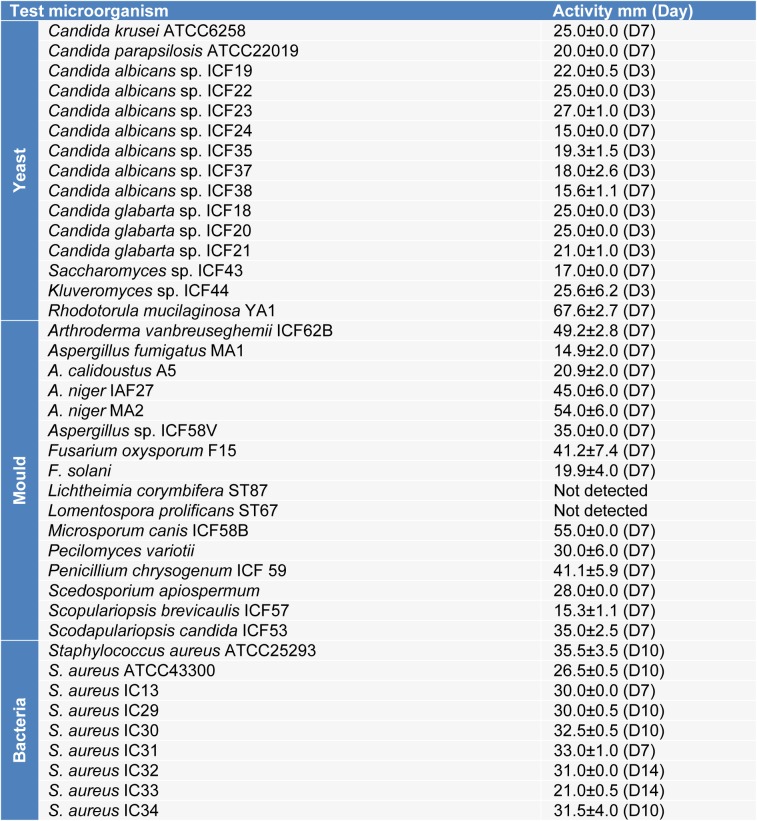
Antimicrobial activity of *Streptomyces* sp. ActiF450

**Table 2:**
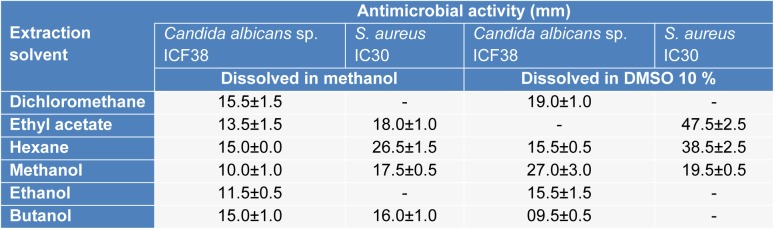
Effect of extraction solvent on antimicrobial activity of ActiF450
